# Dynamics of Recovery of Physiological Parameters After a Small-Sided Game in Women Soccer Players

**DOI:** 10.3389/fphys.2018.00887

**Published:** 2018-07-11

**Authors:** Rafaela B. Mascarin, Vitor L. De Andrade, Ricardo A. Barbieri, João P. Loures, Carlos A. Kalva-Filho, Marcelo Papoti

**Affiliations:** ^1^Post Graduate in Rehabilitation and Functional Performance, Physiotherapy Department, Faculdade de Medicina de Ribeirão Preto, University of São Paulo, Ribeirão Preto, Brazil; ^2^Post Graduate Program in Movement Sciences, Bioscience Institute, Physical Education Department, São Paulo State University “Júlio de Mesquita Filho”, Rio Claro, Brazil; ^3^Post Graduate Program in Physical Education and Sport, School of Physical Education and Sport of Ribeirão Preto, Ribeirão Preto, Brazil

**Keywords:** training, soccer, heart rate variability, muscle damage, hormone, fatigue, recovery, sport science

## Abstract

**Purpose:** Training methods based on small-sided game (SSG) seem to promote physiological and tactical benefits for soccer players as they present characteristics more specific to the game. Thus, the main objective of the present study was to analyze the hormonal, biochemical, and autonomic parameters in an acute manner and the recovery dynamics (up to 72 h after) in a SSG.

**Methods:** Thirteen professional female soccer players participated in the study (18.8 ± 0.8 years, body mass 59.4 ± 6.2 kg, and height 1.68 ± 0.05 m). During and after the SSG session (4 min × 4 min separated by 3 min of passive interval and 120 m^2^ coverage per player), autonomic modulation was analyzed in the time and frequency domains using heart rate variability, and blood samples (5 ml) were collected before (0 h) and after (10 min and 24, 48, 72 h) the SSG for biochemical and hormonal analysis.

**Results:** The SSG induced an increase effect for LF (low frequency) (92,52%; *Very likely increase*) and a decrease effect for HF (high frequency) values (-65,72%; *Very likely decrease*), after 10 min of recovery. The LF/HF increase after 10 min of recovery (386,21%; *Very likely increase*). The RMSSD (square root of the mean squared differences of the successive N–N intervals) and pNN50 (measure of the number of adjacent NN intervals which differ by more than 50 ms) values presented a decrease effect 10 min after SSG (61,38%; *Very likely decrease and*-90%; *Very likely decrease*). The CK (creatine kinase) values presented no changes 10 min after SSG. The LDH (lactate dehydrogenase) values presented an increase effect 10 min after the SSG (19,22%; *Likely increase*). Both testosterone and cortisol concentrations presented the same behavior after SSG, where no alterations were observed with after 10 min (<0,37%; *Most likely trivial*).

**Conclusion:** The SSG promoted significant cardiovascular stress that was restored within the first 24 h of recovery. Parasympathetic parameters continued to increase while sympathetic parameters declined significantly during the 72 h of recovery. In addition, the reduced game did not alter biochemical or hormonal responses during the 72 h.

## Introduction

Decisive actions during an official football match are carried out at maximum intensity over short periods of time (i.e., anaerobic efforts), however, the majority of energy required during a match is supplied by the aerobic metabolism (Jones and Drust, 2007; Hill-Haas et al., 2009a,b; Casamichana and Castellano, 2010). As a result, several training methods, with and without the ball, have been tested (Helgerud et al., 2001; Hoff et al., 2002; Impellizzeri et al., 2006; Little and Williams, 2006; Rampinini et al., 2007; Iaia et al., 2009).

In this sense, different small-sided game have become widely used alternatives, mainly to include actions with the ball, opponents, and specific situations of the game such as defensive or offensive numerical superiority or inferiority (recurring and decisive context in a game of soccer) (Costa et al., 2009). SSGs present specificity, subjecting the participant to the technical, tactical, and physical aspects inherent in soccer practice due to characteristics very close to the formal game (i.e., physical and physiological impact, ball actions, and the presence of opponents and teammates that imply specific situations of the game such as defensive or offensive numerical superiority/inferiority) (Michailidis, 2013). In this way, different SSGs present a high degree of specificity, subjecting the participant to the technical, tactical, and physical aspects inherent in soccer (Owen et al., 2004; Little and Williams, 2006; Jones and Drust, 2007; Michailidis, 2013). By exposing the athletes to a certain level of physical stress, SSGs promote changes in blood lactate concentration, rate of perceived exertion and heart rate, as well as alterations in the autonomic nervous system (Boullosa et al., 2013). One method used to evaluate the autonomic nervous system and its sympathetic and parasympathetic branches is HRV which describes the dynamics of the intervals between consecutive heart beats.

Vanderlei et al. (2009) described that part of the control of the cardiovascular system is performed by the autonomic nervous system and is closely linked to heart rate. Thus, the increase in HR is a consequence of the greater action of the sympathetic pathway and the lower parasympathetic activity. The authors state that irregularities in HRV indicate the heart’s ability to respond to multiple stimuli such as exercise. Thus, the rigorous training programs that professional athletes follow lead to significant changes in the mechanisms of cardiovascular adaptation, improving cardiac function (Francavilla et al., 2018).

After acute physical exercise, HRV can allow easy and non-invasive analysis of the neural control of heart rate, besides being able to measure important modifications in the functioning of the cardiovascular system and its mechanisms of autonomic adjustments (Alonso et al., 1998). The cardiac autonomic modulation index has been used as a marker of the quality of cardiac function, representing a technique that allows the evaluation of risks of sudden cardiac death (Sessa et al., 2018) and also of the stress induced by exercise (Mazon et al., 2013). This analysis is an attempt to avoid states of fatigue, in order to promote adequate recovery, thus optimizing the training (Bricout et al., 2010). Moreover, it presents sensitivity to the effects of the SSG on the autonomic system as observed by Hammami et al. (2016). The study found low parasympathetic reactivation 10, 20, and 30 min after an SSG effort.

In addition to variables related to the cardiovascular system, the determination of injury biomarkers and physiological stress are also frequently used to determine the internal training load (Nakamura et al., 2010; Souza et al., 2010; Coelho et al., 2013; Mazon et al., 2013). Previous studies have shown that both soccer training and formal games can alter plasma concentrations of catecholamines (adrenaline and noradrenaline), cortisol, testosterone, creatine kinase, and lactate dehydrogenase as a consequence of the efforts (Coelho et al., 2011, 2013; Silva et al., 2012, 2014), which could be partially attributed to intermittent repetitions of intense eccentric activation (Ispirlidis et al., 2008).

Different responses are observed between the sexes, mainly in the inflammatory profile (Souglies et al., 2015). Bowtell et al. (2016) investigated the CK response in women with little or no experience of American football during two sessions of different SSGs and found elevated levels of the protein up to 48 h post-game. Ispirlidis et al. (2008) investigated performance, muscle damage, and inflammation during a 6-day recovery period in elite soccer players after a simulated game and found elevated CK and LDH levels up to 96 and 72 h after, respectively, while cortisol levels reached a peak immediately after the game and returned to baseline within the first 24 h of recovery. No change was observed in testosterone levels.

Although it is possible that the delay in HRV recovery after exercise may be indicative of the overall magnitude of the induced stress response, the course of recovery time does not indicate total recovery from the systemic stress response (Seiler et al., 2007). Therefore, simultaneous evaluation of HRV and other markers of stress and fatigue is of utmost importance. Thus, the main objective of the present study was to determine and understand the recovery dynamics of autonomic, biochemical, and hormonal parameters after SSG effort in soccer players.

The SSG seems to be advantage to the training routine, however, little is known about the dynamics of recovery of physiological parameters with this stimulus. Therefore, the main innovative factor of the research was the determination and the understanding of the dynamics of recovery of autonomic, biochemical, and hormonal parameters after the SSG with women soccer players.

## Materials and Methods

### Participants

Thirteen athletes belonging to a professional women’s soccer team participated in the study, who competed in state championships, with minimum experience of 5 years of systematized training, all affiliated to the Brazilian Football Confederation (CBF) [age: 18.8 ± 0.8 years; body weight: 59.4 ± 6.2 kg (Evolution Sanny Professional Precision-Scale); height: 1.68 ± 0.05 m (Sanny Standard Stadiometer); VO_2max_: 36,07 ± 7,50]. All procedures were approved by the University’s Institutional Review Board for Human Subjects (Human Research Ethics Committee) and were conducted according to the Declaration of Helsinki. Athletes were informed about the experimental procedures and risks and signed an informed consent form prior to participation in the study. This study was performed in accordance with international ethical standards (Harriss and Atkinson, 2015).

### Experimental Design

The evaluations were performed 2 months after the competitive period (August, 2017) and all sessions took place on synthetic grass (where the formal games of the team took place) wearing cleats. On the first day, a progressive test (20 m go and back) was performed on the field to determine maximum oxygen consumption (VO_2max_). The SSG was applied on the second day of evaluations, 1 week after the application of the progressive test. All players did not practice any physical activity for 48 h preceding the SSG.

Heart rate variability and HR were evaluated constantly (i.e., prior to, during the SSG, and in the first 30 min and 24, 48, 72 h of recovery). At 0 h, 30 min, 24, 48, and 72 h after the SSG, HRV monitoring was performed for 20 min. Blood samples for biochemical and hormonal analysis were collected prior to, and 5 min and 24, 48, 72 h after the SSG session.

The SSG took place at the team training center in atmospheric conditions of 25–28° C, 40–44% humidity, wind 13 km/h, and atmospheric pressure 1013–1016 hPa (*App The Weather Channel*).

### Progressive Test and Backward Extrapolation Technique

Before the beginning of the tests, the athletes were kept in a seated position for 5 min to determine the baseline of the blood lactate concentration and oxygen consumption (VO_2_). The participants performed 20 m races in the form of go and back on the soccer field. They started the test at an intensity of 8 km/h and increased 1 km/h every 3 min. The intensity of each stage was controlled by sound stimuli and the athletes were instructed to pass the 20 m demarcation lines at each signal. Exhaustion was defined as the player’s inability to continue the test or when she could not complete the 20 m at each beep for three consecutive times.

After each effort, athletes were instructed to breathe immediately into a face mask, connected to a gas analyzer system (VO2000, Medgraphics, EUA). VO_2_ values were log-transformed and plotted against time, which was linearly adjusted. Thus, the y-intercept was considered as VO_2_ at the end of exercise (Montpetit et al., 1981) and assumed as the first point of recovery.

### Small-Sided Game

The coverage area per player was set at 120 m^2^ (Kelly and Drust, 2009; Jastrzebski et al., 2016). The evaluated model was the 4x4; each session lasted 25 min, with 16 min of effort (four efforts of 4 min) and 9 min of passive rest (three rest intervals of 3 min). To perform the evaluations in a staggered way and to respect the minimum interval between evaluations post-SSG, a total of 16 sequential games of 4 min duration and 3 min interval were played. The players warmed up before the start of the SSG with three laps running around the field and short runs with a change of direction for 8 min.

The game consisted of passing the end lines with the ball controlled and possession of the ball was alternated, that is, when a team scored or exceeded the demarcation limits of the game, the ball was quickly returned to the other team. The athletes were motivated by the coaches throughout all games.

### Analysis of Heart Rate Variability (HRV)

The HRV was analyzed pre, 10 min after, and at 24, 48, and 72 h of recovery after the SSG. With the exception of collections 10 min after play, the collections were part of the players’ first daily activity. The players woke up at the training center and went to a pre-determined room for evaluation to begin at 6:30 AM. The HRV was recorded beat-to-beat (RR intervals) by a heart rate monitor – Polar Team^2^ (Polar Kempele^®^, Finland) in a continuous manner and later transmitted to a computer through interface model – IR interface (Polare^®^, Finland) using the Software “Kubios HRV,” for Windows (Polar Electro Oy, Kempele, Finland, 2010).

Heart rate variability was analyzed in the frequency domain: the power of the high frequencies (HF: 0.15–0.40 Hz) and low frequencies (LF: 0.04–0.15 Hz) in normalized units and the LF/HF in ms^2^ (milliseconds). In the time domain, the following indices were used: mean RR (mean of RR intervals), SDNN (standard deviation of all normal RR intervals recorded in a time interval, expressed in ms), RMSSD (square root of the difference between adjacent normal RR intervals in a time interval expressed in ms), and pNN50 (percentage of adjacent RR intervals with duration difference greater than 50 ms) (Task Force of the European Society of Cardiology and the North American Society of Pacing and Electrophysiology, 1996).

### Blood Collection and Analysis

All venous blood collections were performed under the responsibility of an accredited nurse, following all hygiene and asepsis care. Analyzes of the samples were performed by the Clinical Analysis Service (CAS) of the Faculty of Pharmaceutical Sciences of Ribeirão Preto. The athletes were instructed to maintain a 12-h fast, not to practice physical activities, and not to consume alcohol or drinks containing caffeine. While the female athletes were still fasting, 5 mL of blood was collected (7 AM) at moments 0, 24, 48, and 72 h after the SSG in a predetermined room in the training center. The collection 5 min after the game was performed between 10 and 12 AM in a room next to the field where the SSG was played.

For collection and storage of blood samples, BD Vaccutainer^®^ EDTA tubes with separator gel were used (1 Becton Drive, Franklin Lakes, NJ, United States). After collection, the blood was centrifuged for 8 min at 3000 rpm and 8°C and stored at 8°C for further biochemical and hormonal analysis.

For quantification of cortisol and free testosterone, specific radioimmunoassay procedures were used through the IMMULITE/IMMULITE 1000 Total Testosterone and Cortisol Kit (Siemens Medical Diagnostics, Los Angeles, CA, United States). As a marker of muscle damage, CK and LDH were determined with the aid of a specific kit provided by Wiener lab. CK dosing was performed using the optimized UV method (IFCC) in serum. LDH was performed through the optimized UV method (SFBC) in serum.

### Statistical Analysis

The normality of the data was confirmed using the Shapiro–Wilk test, which allowed the description of the variables using mean ± standard deviation. The values observed in each recovery time were compared with baseline values using the Magnitude Based Inferences using the spreadsheets proposed by Hopkins et al. (2009). The effects on HRV, biochemical and hormonal parameters were classified qualitatively as an increase effect, trivial effect or decrease effect. For this, the differences from baseline values were expressed as standardized differences (Cohen’s d) and the smallest standardized change was assumed to be 0.20 (Cohen, 1988). Qualitative inferences were classified as most unlikely (<1%), very unlikely (1–5%), unlikely (5–25%), possibly (25–75%), likely (75–95%), very likely (95–99%), and most likely (>99%). The inference was Unclear when both the increase and the decrease effects were > 5%.

## Results

### Anthropometric and Physiological Characteristics

**Table 1** presents the values referring to the anthropometric characteristics and physiological variables found in the progressive test performed by the players.

**Table 1:** Mean ± standard deviation (SD) values of the anthropometric characteristics and the physiological variables of the players.

Variables	Mean	*SD*
Age (years)	18,80	0,80
Weight (kg)	59,40	6,20
Height (m)	1,70	0,10
BMI (kg/m^2^)	21,00	1,30
LAN (km/h)	11,41	0,39
iVO_2máx_ (km/h)	12,23	0,33
VO_2máx_ (L.min)	3,85	1,38
VO_2máx_ (ml.kg.min)	36,07	7,50

*BMI, body mass index; LAN, anaerobic threshold; iVO_*2max*_, intensity of maximum oxygen consumption; VO_*2max*_, maximum oxygen consumption.*

### Small-Sided Game

The players presented a mean blood lactate concentration ([La]_mean_) of 2.66 ± 0.95 mM at the anaerobic threshold during the incremental test. During the SSG, the [La]_mean_ and %HR_max_ attained were 6.35 ± 2.22 mM and 94.67 ± 0.87%, characterizing the high energy demand in this activity.

### Heart Rate Variability

**FIGURE 1:**
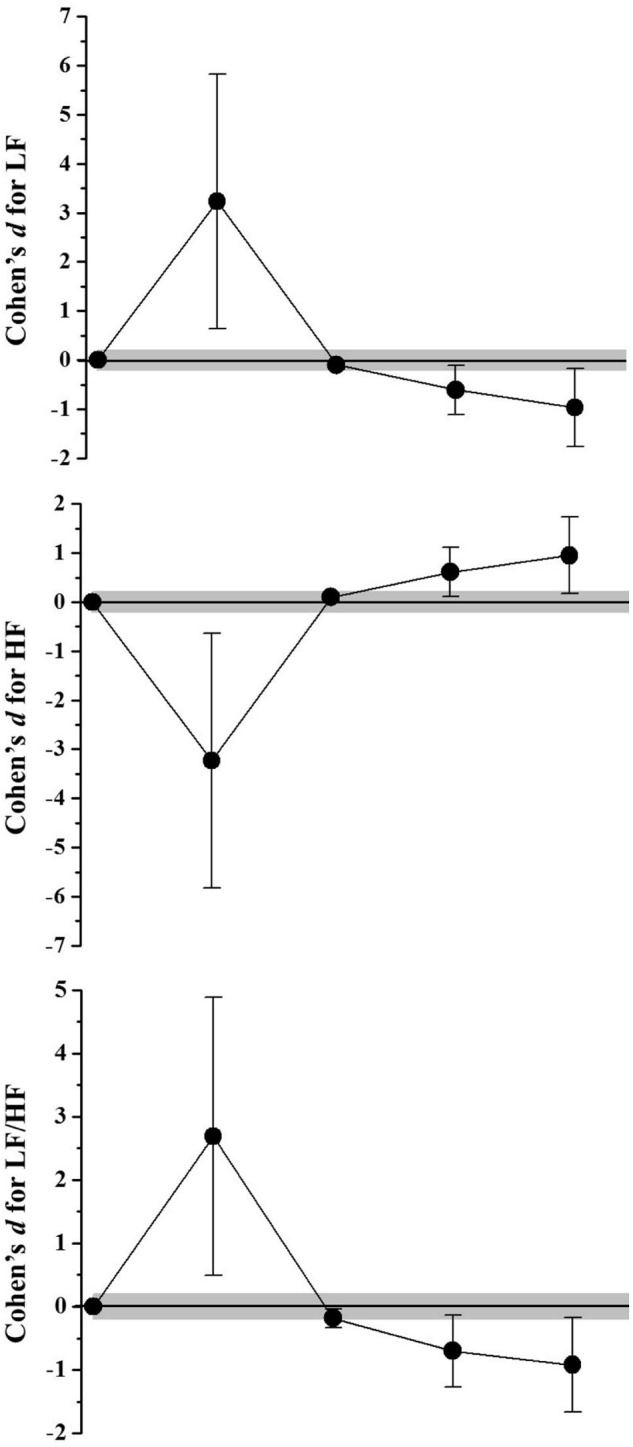
Standardized differences (Cohen’s d) and magnitude-based inference analysis for HRV responses in frequency domain. Chances of effects (decrease/trivial/increase; inference) for Low frequency (LF) values were: after 10 min (2/1/97; Very likely increase), 24 h (0/97/3; Very likely trivial), 48 h (92/8/1; Likely decrease), and 72 h (95/4/1; likely decrease). For high frequency (HF) were: after 10 min (97/2/1; Very likely decrease), 24 h (0/97/3; Very likely trivial), 48 h (1/8/92; Likely increase), and 72 h (1/5/94; likely increase). For LF and HF ratio (LF/HF): after 10 min (2/1/97; Very likely increase), 24 h (42/58/0; Possibly trivial), 48 h (93/6/1; Likely decrease), and 72 h (94/5/1; likely decrease).

**FIGURE 2:**
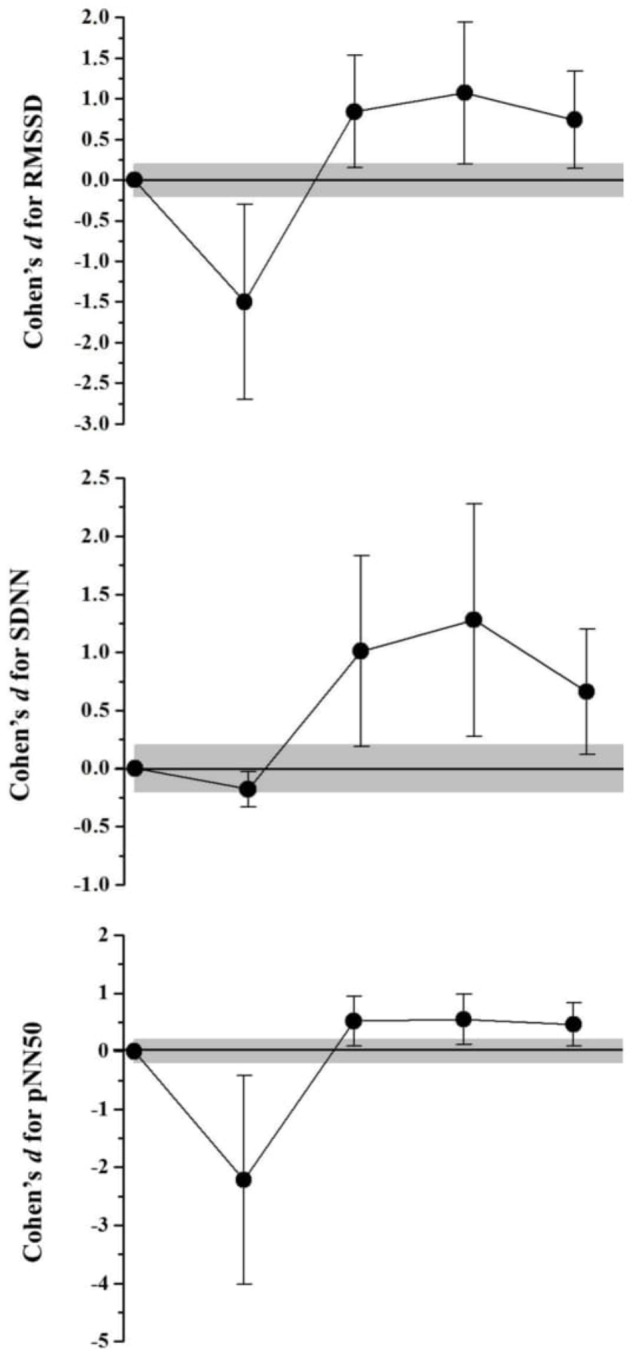
Standardized differences (Cohen’s d) and magnitude-based inference analysis for HRV responses in time domain. Chances of effects (decrease/trivial/increase; inference) for RMSSD values were: after 10 min (96/3/2; Very likely decrease), 24 h (1/5/94; Likely decrease), 48 h (1/4/95; Likely increase), and 72 h (1/6/93; Likely increase). For pNN50 were: after 10 min (97/2/1; Very likely decrease), 24 h (1/9/90; Likely increase), 48 h (1/9/90; Likely increase) and 72 h (1/12/88; Likely increase). For SDNN: after 10 min (40/60/0; Possibly trivial), 24 h (1/4/95; Likely increase), 48 h (1/3/96; Very likely increase), and 72 h (93/7/1; Likely increase).

The HRV responses were demonstrated in **Table 2**. In the frequency domain (**Figure 1**), SSG induced an increase effect for LF (92,52%; *Very likely increase*) and a decrease effect for HF values (-65,72%; *Very likely decrease*), after 10 min of recovery. Both LF and HF returned to baseline values after 24 h (<2,13%; *Very likely trivial* effect) and presented effects related to the autonomic adaptation after 48 h (*Likely decrease* for LF and *Likely increase* for HF), which was maintained after 72 h. The LF/HF increase after 10 min of recovery (386,21%; *Very likely increase*), returned to baseline values after 24 h (13,44%; *Possibly trivial*) and decrease after 48 and 72 h of recovery (-53%; *Likely decrease*). In the time domain (**Figure 2**), the RMSSD values presented a decrease effect 10 min after SSG (61,38%; *Very likely decrease*) but showed an increase effect from 24 h of recovery (>57,04%; *Likely increase*). The same behavior was observed for pNN50, where a decrease effect occurred after 10 min (-90%; *Very likely decrease*), which was followed by an increase from 24 h of recovery (>15,28%; *Likely increase*). Although the SDNN values demonstrated no alterations 10 min after the SSG (-13,52%; *Possibly trivial*), an increase effect was also observed from 24 h of recovery (>49,03%; *Likely increase*).

**Table 2:** Descriptive values for the heart rate variability parameters.

		Recovery			
	Baseline	10 min	24 h	48 h	72 h
RR (ms)	1083,32 ± 165,55	739,63 ± 87,57	1150,16 ± 205,17	1097,32 ± 209,01	1137,35 ± 192,49
LF (un)	41,49 ± 16,32	79,88 ± 6,08	42,38 ± 19,65	34 ± 12,95	27,95 ± 12,37
HF (un)	58,43 ± 16,31	20,03 ± 6,08	57,56 ± 19,64	65,92 ± 12,97	71,77 ± 12,42
LF/HF (ms^2^)	0,91 ± 0,85	4,43 ± 1,66	1,03 ± 1,05	0,57 ± 0,33	0,43 ± 0,25
RMSSD (ms^2^)	61,68 ± 32,62	23,82 ± 12,89	96,87 ± 77,96	137,65 ± 129,58	102,33 ± 89,34
pNN50 (%)	40,53 ± 24,75	4,05 ± 4,55	46,72 ± 27,78	50,23 ± 32,36	50,34 ± 23,95
SDNN (ms^2^)	63,62 ± 27,80	55,01 ± 31,97	94,81 ± 51,67	122,98 ± 58,85	40,53 ± 24,75

*RR, interval beat-to-beat; LF, low frequency; HF, high frequency; LF/HF, low frequency/high frequency; RMSSD, square root of the mean squared differences of the successive N–N intervals; SDNN, standard deviation of the normal-to-normal intervals; pNN50, proportion of interval differences of successive N–N intervals greater than 50 ms.*

### Biochemical and Hormonal Examinations

**FIGURE 3:**
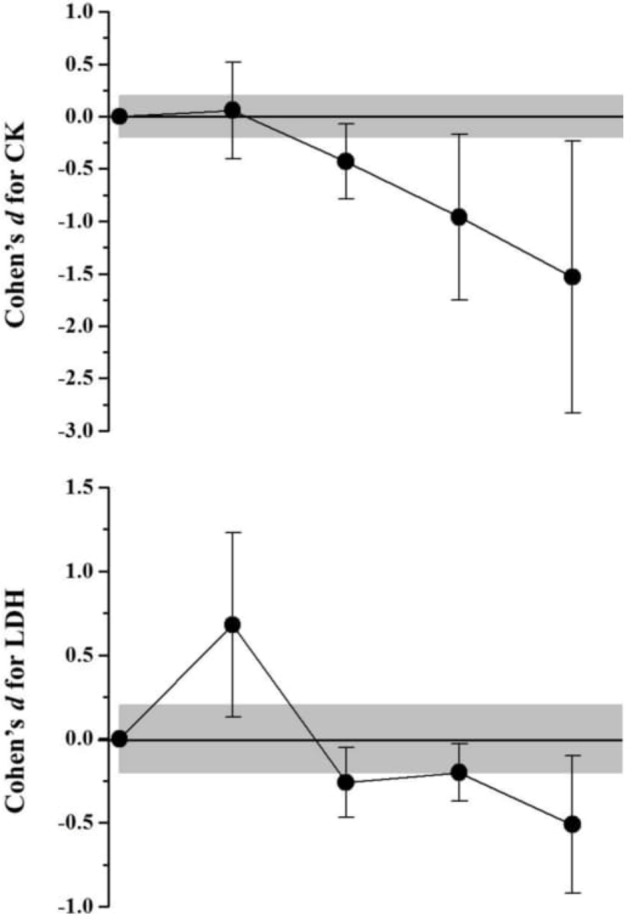
Standardized differences (Cohen’s d) and magnitude-based inference analysis for muscle damage markers. Chances of effects (decrease/trivial/increase; inference) for CK were: after 10 min (0/100/0; Most likely trivial), 24 h (87/13/0; Likely decrease), 48 h (95/4/1; Likely decrease), and 72 h (96/3/1; Very likely decrease). For LDH were: after 10 min (1/7/92; Likely increase), 24 h (69/31/0; Possibly decrease), 48 h (51/49/0; Possibly decrease), and 72 h (89/10/1; Likely decrease).

**FIGURE 4:**
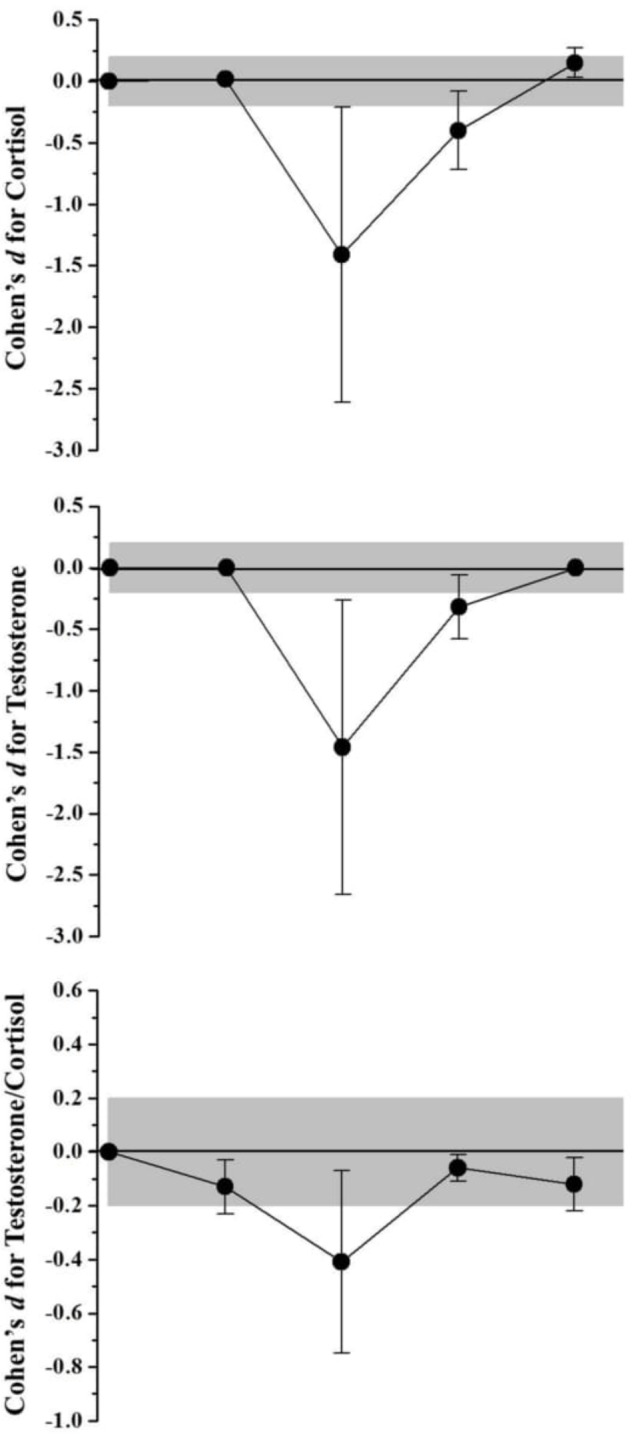
Standardized differences (Cohen’s d) and magnitude-based inference analysis for hormonal variables. Chances of effects (decrease/trivial/increase; inference) for cortisol values were: after 10 min (0/100/0; Most likely trivial), 24 h (96/3/1; Very likely decrease), 48 h (85/15/0; Likely decrease) and 72 h (0/77/23; Likely trivial). For testosterone were: after 10 min (0/100/0; Very likely decrease), 24 h (96/3/2; Very likely decrease), 48 h (79/21/0; Likely decrease), and 72 h (0/100/0; Most likely trivial). For testosterone and cortisol ratio (Testosterone/Cortisol): after 10 min (12/88/0; Likely trivial), 24 h (86/14/0; Likely decrease), 48 h (0/100/0; Most Likely trivial), and 72 h (8/92/0; Likely trivial).

Biochemical and hormonal responses were presented in **Table 3**. **Figure 3** shows the muscle damage values obtained before and during recovery after SSG. The CK values presented no changes 10 min after SSG (2,72%; *Most likely trivial*) and decrease progressively from 24 h of recovery (> -19,59%; *Likely decrease* until 48 h and *Very likely decrease* at 72 h). Although the LDH values presented an increase effect 10 min after the SSG (19,22%; *Likely increase*), these concentrations decrease progressively from 24 h of recovery (> -7,68%; *Likely decrease* until 48 h and *Very likely decrease* at 72 h). Both testosterone and cortisol concentrations presented the same behavior after SSG (**Figure 4**), where no alterations were observed with after 10 min (<0,37%; *Most likely trivial*), an decrease effect occurred after 24 h (> -32,65%; *Very likely decrease*) and 48 h >8,92%; *Likely decrease*), with the return to the baseline values after 72 h of recovery (< -0,09%; *Most likely trivial* for testosterone and *Likely trivial* for cortisol).

**Table 3:** Descriptive values for the muscle markers and hormonal variables.

		Recovery			
	Baseline	10 min	24 h	48 h	72 h
CK (U/L)	231,62 ± 127,44	237,92 ± 95,09	186,23 ± 81,61	147,77 ± 47,06	108,38 ± 33,68
LDH (U/L)	397,38 ± 127,87	473,77 ± 98,29	366,85 ± 107,13	374,62 ± 96,51	343,85 ± 84,05
Cortisol (ug/dL)	16,30 ± 3,66	16,36 ± 3,35	10,98 ± 3,87	14,85 ± 3,67	16,85 ± 3,68
Testosterone (ng/dL)	32,44 ± 9,88	32,49 ± 12,39	19,11 ± 8,32	29,51 ± 8,42	32,41 ± 10,56
T/C	2,11 ± 0,81	2,01 ± 0,70	1,79 ± 0,72	2,06 ± 0,75	2,01 ± 0,83

*CK, creatine kinase; LDH, lactate dehydrogenase; T/C, testosterone/cortisol.*

## Discussion

The investigation of the autonomic, biochemical, and hormonal parameters pre- and post-SSG, demonstrate that the stimulus promoted a break in the organic homeostasis of soccer players. In this sense, was determined and monitored the dynamics of recovery of autonomic, biochemical, and hormonal that promote specific and desired adaptations in women soccer players in the training routine.

The temporal course of cardiac autonomic recovery reflects the restoration of cardiovascular homeostasis, which is an important component of general recovery (Stanley et al., 2013). Thus, HRV indices may be useful for monitoring the effects of soccer training as they are sensitive to periods of stress and recovery (Bara-Filho et al., 2013). In relation to this, the findings of Boullosa et al. (2012), with male and female Spanish soccer players, suggest that a higher baseline HRV may allow greater use of autonomic resources for responses of soccer players to stress. Dutra et al. (2013) investigated baseline HRV indices in women divided into three groups according to aerobic capacity and found values similar to those of the present study for RR, LF, HF, and the LF/HF ratio. In a study conducted with trained and highly trained runners (Seiler et al., 2007), baseline values for all autonomic indices corroborate with the data of the present study. Similar values were also found in soccer players during the pre-season (Oliveira, 2012).

The results of the present study demonstrate a high mean RR and HF (parasympathetic predominance index) pre-game, followed by a significant decrease 10 min after the SSG. The values of HF pre- SSG corroborate with a study conducted with female professional basketball players (Messina et al., 2012). The authors suggest that a lower resting heart rate is a consequence of high vagal tone due to the training effect. In relation to the LF and LF/HF ratio (indices related to the predominance of the sympathetic component action on the heart), low pre-SSG means were observed followed by a significant increase in the first 10 min of recovery. These results reflect an increase in sympathetic stimulation or an attenuated parasympathetic modulation mitigated by the SSG (Boullosa et al., 2012) in order to bring the ANS to a stress condition and consequently, low HRV values that are attributed to a decrease in the efferent vagal tonus and a lower β-adrenergic response capacity (Dong, 2016). That is, during exercise, with the increase in HR, autonomic dysfunctions occur such as vagal inhibition and increased sympathetic activation (Buchheit et al., 2009). This post-exertion behavior has been reported in several studies with varied efforts in soccer among young trained individuals, untrained individuals, players, and elite players (Bricout et al., 2010; Boullosa et al., 2012, 2013; Bara-Filho et al., 2013; Dellal et al., 2015; Flatt et al., 2016, 2017; Hammami et al., 2016).

When the recovery data were observed 24 h after exercise, it was observed that HRV values returned to baseline and continued to decrease (LF and LF/HF) or increase (RR, HF, RMSSD, pNN50, and SDNN) in the following hours. This is due to parasympathetic cardiac reactivation. We emphasize the decrease in LF and a significant increase in HF found at 72 h in relation to the pre-game and 24 h recovery moments. This result may be associated with the recommendation not to practice any physical activity for only 48 h preceding the test. It is possible that if there had been a pause in the training sessions in the 72 h that preceded the SSG, the values found in the pre-analysis would not show a significant difference in relation to the 72 h moment. Another hypothesis is based on the fact that the SSG and other collections performed in the study may have altered the daily autonomic control of the players. The participants in the study of Boullosa et al. (2012) presented significantly lower HRV before and after a football match compared to the day of rest. The authors state that concern or mental preparation for the soccer game may lead to an increased sympathetic response and/or attenuated parasympathetic modulation, resulting in lower player HRV.

Based on the results, the players demonstrated significant cardiovascular stress during the SSG with decreased cardiac autonomic control, evidenced in the first minutes of recovery (10 to 30 min) in relation to the pre-game. Previous studies with a simulated formal game in soccer players observed low HRV in up to 10 h of recovery (Boullosa et al., 2013). In contrast, Seiler et al. (2007) in a study with highly trained runners observed recovery at approximately 120 min post-exercise, regardless of the intensity of the training. However, Stanley et al. (2013) demonstrate that the time required for complete autonomic cardiac recovery after a single aerobic training session is up to 24 h after low-intensity exercise, 24–48 h after moderate exercise, and at least 48 h after high intensity exercise. However, the authors suggest that individuals with higher fitness are more resistant to training stress and require less time to recover due to lower variations and faster recovery of cardiac parasympathetic activity after exercise. In the present study, although the SSG was an intense aerobic activity (92.7–94.77% HRmax), cardiovascular autonomic recovery occurred after 24 h.

The baseline plasma CK concentrations of the present study are close to those found by Coelho et al. (2011) when evaluating soccer players of the first division of Brazilian soccer. The authors evaluated the team throughout the training period and, therefore, values close to 300 U/L are expected during the season. These values are also similar to those of Zoppi et al. (2003), Ascensão et al. (2008), and Souglies et al. (2015). In contrast, Lazarim et al. (2009) found higher resting values (493 U/L) and Andersson et al. (2007), lower values (158 ± 33 U/L) when analyzing protein concentrations in professional soccer players. The latter author, however, did not report the interval between CK collection and team training. In relation to LDH, Bezerra et al. (2016) found resting values close to those of the present study and reported that the interval between the final training and collections was 24 h, suggesting that the values found were influenced by the daily training. Ispirlidis et al. (2008), when evaluating professional soccer players, pre- and post-game, found CK and LDH resting values below 200 U/L, however the author states that the athletes did not practice any strenuous activity for 7 days before and after the game. In the present study, the players stopped training 48 h preceding the SSG, and for this reason it was possible to observe a significant reduction in CK and LDH at 48 h in relation to at 5 min and 24 h of recovery.

Despite resting values close to those reported in the literature, there was no significant increase in CK in the 72 h of recovery in relation to rest. On the other hand, it was possible to observe a significant increase in LDH soon after (10 min) the SSG. Observing the other results, this increase is associated with a decrease in the O_2_ demand in the muscle and, therefore, intensification in the lactate formation in order to provide energy for muscular action.

No studies were found that assessed muscle damage in response to an SSG with female soccer players. Bowtell et al. (2016) analyzed the CK response using soccer SSGs in untrained women, however, the rest values presented were much lower (69 ± 23 U/L) than in the present study. After the SSG the authors found values significantly higher than pre-game, with the peak at 48 h of recovery (108 ± 39 U/L). The literature is vast concerning responses to game stimuli and muscular damage in soccer players (Andersson et al., 2007; Ascensão et al., 2008; Ispirlidis et al., 2008; Coelho et al., 2011; Souglies et al., 2015). Thus, it can be concluded that although the practice of the SSG chosen may be intense, it does not impose stimuli that produce muscular stress when compared to the formal game, probably because of its short duration.

By monitoring the quantitative changes in hormones with anabolic and catabolic properties, such as testosterone and cortisol, it is possible to identify a momentary catabolic state (Mazon et al., 2013). Several studies have reported the behavior of these hormones against stimuli from formal male and female soccer games (Ispirlidis et al., 2008; Oliveira et al., 2009; Maya et al., 2016). Haneishi et al. (2007), in addition to evaluating the post-game responses, analyzed the cortisol responses after training of 105 min. The authors found that the post-game cortisol response was 250% higher than the post-training values, which did not present any significant differences in relation to the pre-training evaluation. Competitive events (i.e., games) are more likely to generate acute hormonal responses than routine training activities (e.g., SSGs), as they promote an early increase in cortisol levels to prepare the individual for action (Oliveira et al., 2009). In the present study, there was no significant increase in cortisol or testosterone during the 72 h of recovery after the SSG. Waal (2017) evaluated the acute endocrine responses of soccer players in an SSG close to the model proposed in the present study and also found no significant difference after the SSG in relation to rest. This appears to be the only study to evaluate hormonal responses from stimuli using SSGs. The author also concludes that training based on SSGs or unofficial (i.e., friendly) matches does not seem to produce the same significant hormonal responses to the stimulus as the competitive environment. Consequently, no significant alterations were observed in the T/C ratio.

It can be concluded that the athletes presented cardiovascular stress during the SSG with reduced cardiac autonomic control, evidenced in the first minutes of recovery. The parasympathetic cardiac reactivation was reestablished after 24 h although the values at 72 h still demonstrated a significant reduction. However, although the physical requirements related to the SSG caused a decrease in the autonomic parameters, the hormonal and muscle damage markers were not altered.

The limitation of the present study was the relatively small number of participants. The study evaluated 13 players, however, a total of 23 players took part in the study to make the ideal scheduling possible in the participation in each of the SSG efforts (fundamental aspect so that the collection moments are met post-SSG for each player) besides the precaution related to possible injuries from the SSG. Nevertheless, the study offers valuable insights into the SSG among women soccer players.

Further studies should be devoted to verifying the influence and recovery time required for autonomic, neuromuscular, inflammatory, and hormonal parameters using generic training methods (e.g., interval aerobic training, intermittent high-intensity training) which seek improvement in aerobic fitness and game performance in male and female amateur and professional soccer players. In addition, new efforts should be directed in an attempt to simulate competitive scenarios using SSGs and generic training methods.

As a practical implications, it is important that high performance coaches simulate competitive practice environments in order to make training, based on internal loads, as close as possible to the context and physiological demand experienced during a formal competitive football match. Thus, the understanding and monitoring of certain stress markers during the season could contribute to the systematization and optimal control of individual training loads in an attempt to minimize the onset of the fatigue process and enhance performance of the athletes.

## Author Contributions

RM, VDA, RB, CK-F, and MP: conceived and designed the experiments. RM and VDA: performed the experiments. RM, VDA, and RB: analyzed data. RM, VDA, RB, and CK-F: contributed materials and analysis tools. RM, VDA, JL, CK-F, and MP: wrote the paper.

## Conflict of Interest Statement

The authors declare that the research was conducted in the absence of any commercial or financial relationships that could be construed as a potential conflict of interest.

## Abbreviations

AM: before midday
C: cortisol
CK: creatine kinase
HF: high frequency
HR: heart rate
HRV: heart rate variability
LDH: lactate dehydrogenase
LF: low frequency
pNN50: proportion of interval differences of successive N–N intervals greater than 50 ms
RMSSD: square root of the mean squared differences of the successive N–N intervals
RR: interval beat-to-beat
SDNN: standard deviation of the normal-to-normal intervals
SSG: small-sided game
T: testosterone
VO_2_: oxygen consumption
VO_2MAX_: maximum oxygen consumption
